# “University stress” exploring the potential impact of an immersive art experience on a college campus

**DOI:** 10.3389/fpsyg.2025.1592649

**Published:** 2025-11-12

**Authors:** Lyn Godley, C. Virginia O’Hayer, Raegan Davis, Emily Wakschal, Chelsi Nurse, Wendy Ross, Abigail Spraker, Nate Godshall, Rosemary Frasso

**Affiliations:** 1 School of Design and Engineering, Jefferson Center of Immersive Arts for Health, Thomas Jefferson University, Philadelphia, PA, United States; 2 Department of Psychiatry and Human Behavior, Thomas Jefferson University, Philadelphia, PA, United States; 3 Jefferson College of Populations Health, Thomas Jefferson University, Philadelphia, PA, United States; 4 Center for Autism and Neurodiversity, Thomas Jefferson University, Philadelphia, PA, United States; 5 School of Design and Engineering, Department of Industrial Design, Thomas Jefferson University, Philadelphia, PA, United States; 6 Center for Research in Medical Education and Health Care, Sidney Kimmel Medical College, Thomas Jefferson University, Philadelphia, PA, United States

**Keywords:** art and wellbeing, immersive art experiences, mental health benefits, multisensory engagement, stress reduction, college student wellbeing

## Abstract

There is growing interest in the effects of immersive art on wellbeing, which engages multiple senses and facilitates deeper engagement. University settings, particularly during high-stress periods like exams, are increasingly emphasizing mental health interventions to combat rising rates of anxiety, depression, stress, and suicidal ideation. This study investigates the potential of a fully immersive art experience to positively impact mood and reduce stress in a university setting. Pre- and post-visit questionnaires, including the Positive and Negative Affect Schedule (PANAS) survey, were administered to assess emotional changes. Additional open-ended questions provided qualitative feedback. Results showed statistically significant decreases in 12 negative affect scores and increases in 9 positive affect scores. Overall PANAS scores increased by a median of 6 points, indicating improved mood post-experience. Qualitative data highlighted the exhibit’s calming and restorative effects; many participants suggested amending the availability of such installations to a year-round schedule, in order to further promote student mental health.

## 1 Introduction

Exposure to art has been shown to produce short-term improvements in emotional state. For example, Bolwerk et al. (2014) found that engaging with visual art increased psychological resilience and enhanced positive affect. Cupchik et al. (2009) demonstrated that aesthetic engagement with art elicited greater emotional resonance compared to non-art stimuli, suggesting that art uniquely enhances emotional processing in ways that elevate mood. Cotter and Pawelski (2022) reported that engagement with art museums was associated with greater cheerfulness, happiness, uplift, and engagement, while Cuypers et al. (2012) found that frequent cultural participation correlated with lower anxiety and better overall health. Other studies extend these findings, showing that visual art can activate brain circuits related to pleasure and intrinsic motivation (Fagioli et al., 2025), reduce stress (Trupp et al., 2025), and increase both positive affect and social connectedness (Igdalova et al., 2025). Daykin et al. (2008) further notes that the arts contribute to wellbeing, provide reassurance, and help foster identity. Chatterjee and Vartanian (2016) proposed that the “aesthetic triad” integrates sensory–motor, cognitive–meaning–making, and emotional systems, offering a framework for how art sharpens focus and encourages reflective thought. Reviews by Fancourt and Finn (2019) and Shim et al. (2021) highlight the arts’ role across the lifespan in preventing ill health, promoting health, and supporting treatment and recovery, with Fancourt and Finn (2019) emphasizing that cultural participation lowers risk of depression and anxiety, while Law et al. (2021) showed that art-based interventions reduce depressive symptoms among university students.

Much of this research has focused on traditional, static art forms such as painting, prints, and sculpture. More recently, scholars have begun to examine how new media art—including immersive experiences—affects health and wellbeing, with promising results. Chirico et al. (2017) found that immersive experiences significantly increased awe, presence, and parasympathetic activation. Croghan et al. (2022) demonstrated reductions in stress among healthcare providers and individuals with PTSD, while Difede et al. (2007) supported similar outcomes. Hadavi et al. (2022) reported that virtual art exhibits increased positive affect and reduced negative affect, indicating overall mood improvement. Immersive and interactive art forms may amplify these benefits by fostering deeper engagement, inducing relaxation, and enhancing empathy. Han et al. (2024), for instance, showed that immersive light installations promoted psychological restoration in urban public settings. Immersive art, unlike traditional art where viewers remain outside the work, places audiences within a multisensory environment that can alter perceptions of time, space, self, and social connection (Mäcklin, 2019). These installations often combine sight, sound, and touch (Sommer et al., 2020; Spallazzo and Ceconello, 2024; Yin and Jin, 2022), producing a cognitive, emotional, and physical suspension from ordinary reality (Spallazzo and Ceconello, 2024; Turner et al., 2016).

Despite these encouraging findings, evidence remains uneven related to the impact of exposure to art, in any form, on mental wellbeing. Trupp et al. (2025), in a systematic review, noted convergent support for eudaimonic outcomes (such as self-reflection and personal growth), but highlighted inconsistent findings regarding hedonic outcomes (positive mood, stress reduction).

The COVID-19 pandemic highlighted the importance of addressing mental health, particularly on college campuses where stress among students continues to rise. The 2021–2022 Healthy Minds Survey documented the severity of the campus mental health crisis, gathering data from approximately 90,000 students across 133 U.S. campuses. Nearly half (44%) of respondents reported depressive symptoms, 37% experienced anxiety, and 15% reported suicidal ideation (Lipson et al., 2023). According to the National College Health Assessment, almost three-quarters of students reported moderate or severe psychological distress (National College Health Assessment, American College Health Association, 2021).

Educational leaders are prioritizing mental health initiatives to foster safe, supportive environments that encourage learning and personal growth. Stress on campuses often follows a cyclical pattern, with certain time periods being more challenging than others. Many universities have implemented stress-reduction interventions to address the growing mental health concerns of learners, faculty, and staff (Fernández et al., 2016). On-campus immersive art installations are one such intervention. However, little work has been done focusing on the potential impact of exposure to immersive art on learners and other members of a college community.

The current pilot study sought to examine the impact of exposure to an immersive art installation on mood and stress among university students, faculty, and staff. The installation was open to the college community at the end of the semester, when academic stress, which has been shown to negatively impact mental health in students, is often high (Li and Lin, 2003; Eisenberg et al., 2009; Barbayannis et al., 2022; Špiljak et al., 2022). For the purpose of this study, stress is operationalized as situationally based and short-term within the context end-of-term/exam season. Participants were provided with a measure of *in vivo* negative affectivity pre and post art instillation viewing.

It was hypothesized that self-reported level of various negatively valanced affect terms, including distress, upset, guilt, fear, shame, nervousness and irritability would decrease after experiencing the immersive installation. It was also hypothesized that self-reported level of various positively-valanced emotions, including wonder, connection, relaxation, pride, energy, and calm would increase after the installation.

## 2 Methods

### 2.1 Setting

**Figure 1:**
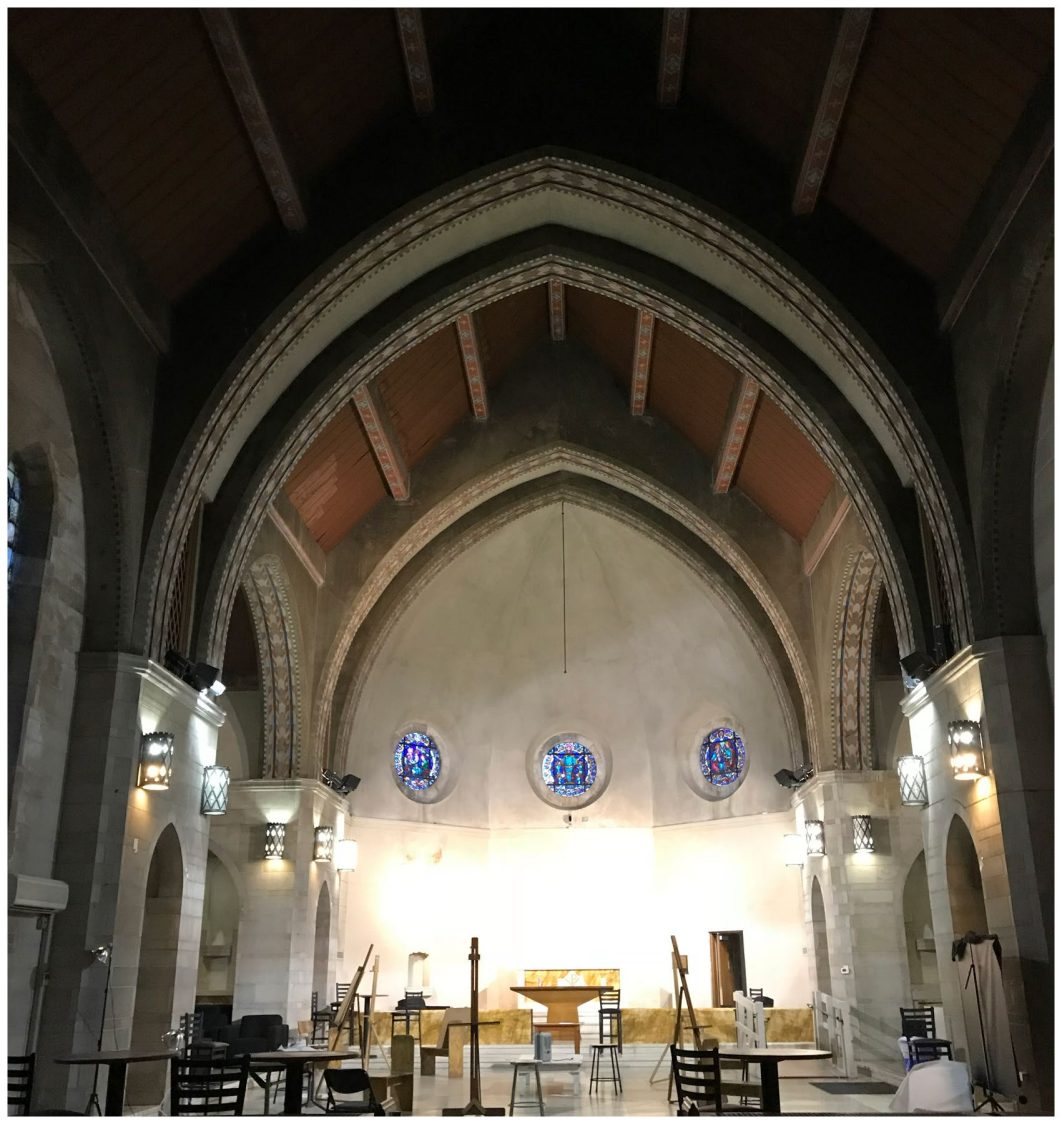
Ravenhill Chapel interior prior to event. Image credit Lyn Godley.

**Figure 2:**
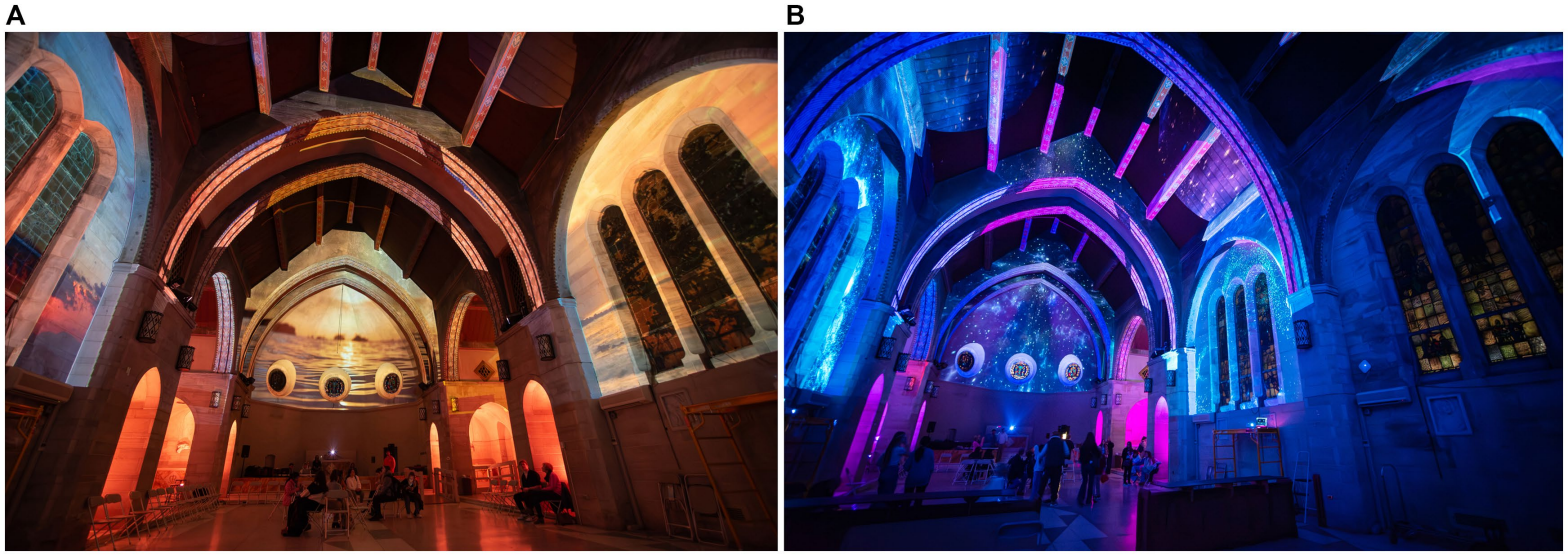
Ravenhill Chapel as an immersive experience [**(A)** sunset imagery, **(B)** galaxy imagery]. Image credit ©Thomas Jefferson University Photography.

**Figure 3:**
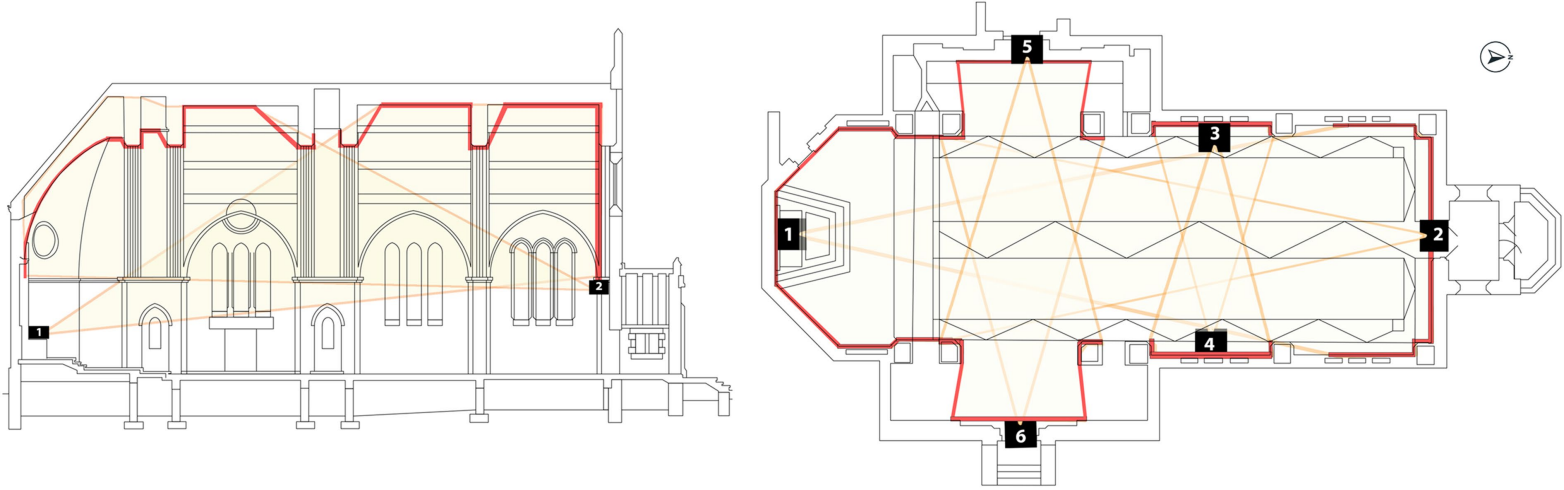
Schematic and floorplan of Ravenhill Chapel showing projector placement. Image credit Lyn Godley and Olivia Dec.

At Thomas Jefferson University in Philadelphia, PA, students enrolled in the undergraduate *Light as Public Experience* course designed and installed an immersive art exhibit. This installation took place in a large open space with no fixed seating, a former campus chapel (Ravenhill Chapel, Figure 1) housed on what is referred to as the East Falls Campus. Students conducted extensive literature review and analyses supporting the selection of imagery and music as well as the tempo, timing, and pairing of images with music passages during the installation. Images on display were chosen with the goal of creating a relaxing, positive environment. For instance, serene sunsets were paired with images of gently crashing waves to recreate the feeling of self-awareness, stress reduction, and relaxation that nature can have on a person (Bell et al., 2015; Nichols, 2014; White et al., 2010). The music was chosen to complement the imagery and was slowed to a meditative pace to connect rhythm with respiratory pattern as described by Haas et al. (1986), encouraging the viewer to surrender to the experience (Spallazzo and Ceconello, 2024). The experience was looped on a 20-min set using projection mapping, and included five thematic videos of jellyfish, sunsets, waves, ink drops, and stars to create a 360-degree visual experience (Figures 2, 3). Additional details about the design and installation can be found in Appendix A. The installation was open to the university community for three evenings from 6:30 p.m. to 9:00 p.m. immediately preceding final examination week in the fall of 2023. Information and invitations about the installation were shared across campus through flyers and word-of-mouth.

### 2.2 Data collection

This pilot study was approved by the Institutional Review Board at Thomas Jefferson University. The team employed a convergent parallel mixed methods design to gather both quantitative and qualitative data by administering pre- and post-visit questionnaires to a convenience sample of people who experienced the immersive art installation. Information about the study was posted on the entryways leading to the installation (including that participation is voluntary). Visitors to the installation who were members of the Jefferson community (students, staff and faculty) were invited to participate in the study upon entering the venue. Participants had to be 18 years of age or older and able to read and write in English. If they agreed, a QR code was provided which was linked to a consent document, followed by the Positive and Negative Affect Schedule, Short Form (PANAS-SF; Watson et al., 1988). The sampling approach did not lend itself to *a priori* power analysis.

The PANAS-SF is one of the most widely used scales to measure mood or emotion (Watson et al., 1988). This brief scale is comprised of 20 items, with 10 items measuring positive affect (e.g., excited, inspired) and 10 items measuring negative affect (e.g., upset, afraid) over a selected period of time in a 5-point Likert scale format. Composite PANAS score is the sum of all positive affect scores with negative affect scores subtracted. Our analysis elected the instrument’s ‘moment’ time instruction, asking respondents about their affect at the exact time of response. Importantly, the initial validation of the PANAS included studies examining intraindividual variation in mood over short timescales, measurements being taken daily or in 3-h intervals (Clark and Watson, 1988; Watson, 1988; Watson et al., 1988). These investigations showed the PANAS having strong relationships with recent social activity and momentary perceived stress. A broad review of measures for anxiety showed that the PANAS NA measure and fear facet had similar sensitivity and usefulness to other anxiety measures (e.g., STAI, POMS) in measuring intra-individual change before and after experimental manipulations in 33 studies (Rossi and Pourtois, 2012). Multiple analogues to our use of the PANAS are found in the literature, including measuring the differential effect of a nature walk over watching a video of nature or walking on a treadmill immediately before the interventions, immediately after the interventions, and after a cold pressor task to induce stress (Olafsdottir et al., 2020).

Additionally, the respondents were asked about three emotions unmeasured by PANAS (wonder, connectedness, and relaxation) in the same Likert style and to indicate their energy level with any number of four binary checkboxes (tired, energized, rejuvenated, and calm). Finally, respondents were asked to answer open-ended questions aimed at capturing perspectives on the value of their experience. Visitors were asked to complete a second identical battery of questionnaires immediately after visiting the venue. Paper copies were available if needed (Appendix A).

### 2.3 Analyses

Responses to the PANAS-SF were summarized and pre-visit and post-visit comparisons were made. Owing to the ordinal nature of the PANAS-SF survey and the sample size, we opted to perform a series of unidirectional Wilcoxon signed-rank tests to gauge the impact of the installation on attendees’ mood (Table 1).

**Table 1:** Wilcoxon signed-rank test results.

Participant-generated sentiments	Pre-test median	Post-test median	Mean difference	*p-*value
Left tailed				
Wonder	2	4	+1.18	<0.0001
Connection	3	3	+0.37	<0.0001
Relaxation	2	4	+1.81	<0.0001
Interest	4	4	+0.13	0.065
Strength	3	3	+0.08	0.195
Enthusiasm	3	3	+0.23	0.007
Pride	3	3	+0.33	<0.0001
Inspiration	3	4	+0.93	<0.0001
Determination	3	3	+0.33	0.001
Attentiveness	3	3	+0.12	0.150
Activeness	3	3	−0.11	0.892
Energy^a^	0	0	+0.01	0.362
Rejuvenation^a^	0	0	+0.39	<0.0001
Calm^a^	0	1	+0.35	<0.0001
PANAS Sum	12	18	+7.01	<0.0001
Right tailed				
Distress	2	1	−0.43	0.001
Excitement	3	3	−0.19	0.026
Upset	1	1	−0.55	<0.0001
Guilt	1	1	−0.42	<0.0001
Scaredness	1	1	−0.35	<0.0001
Hostility	1	1	−0.22	<0.0001
Irritability	2	1	−0.82	<0.0001
Alertness	3	3	−0.18	0.063
Shame	1	1	−0.31	<0.0001
Nervousness	2	1	−0.69	<0.0001
Jitteriness	2	1	−0.90	<0.0001
Fear^a^	1	1	−0.38	<0.0001
Tiredness^a^	1	0	−0.39	<0.0001

a Binary question.

Responses to the open-ended questions were brief and did not lend themselves to in-depth thematic analysis. Therefore, the team employed Rapid Qualitative Analysis (RQA), a pragmatic approach that involves summarizing responses and organizing them into meaningful categories. RQA is commonly used when open-ended survey responses are concise but contain valuable insights that warrant structured interpretation (Kowalski et al., 2024).

## 3 Results

Study team members (CVO’H, RF, others) attempted to approach every attendee prior to entering the installation, to provide information about the pilot study and encourage participation. Almost all attendees agreed to take part. A total of 214, participants consented and 155 individuals completed both the pre-visit and the post-visit questionnaires (there were 53 pretests without competed posttests this group did not differ from those who completed both surveys). Six young adults who self-identified, or were identified by a family member, as part of a neurodiverse community; results from their surveys were excluded from the analysis and will be reported separately. Some 63.63% were students who attended the exhibit the final week of the term, a time when exams and concern about grades are typically associated with elevated acute stress levels among students (Špiljak et al., 2022).

The remaining attendees were members of the campus community (faculty and staff). On average, respondents spent 38 min engaging with the immersive art installation. In total, these paired nonparametric tests revealed a statistically significant decrease in 1 different negative affect scores after individuals were exposed to the immersive art installation and an increase in nine different positive affect scores, as well as an increase in overall PANAS-SF score (*M* difference = +7.01, *p* < 0.0001). Most relevant to our hypothesis that the installation would lower stress and promote a sense of calm, attendees reported feeling less distressed (*M* difference = −0.43, *p* < 0.0001), less scared (*M* difference = *−*0.35, *p* < 0.0001), less afraid (*M* difference = *−*0.38, *p* < 0.0001), less upset (*M* difference *= −*0.55, *p* < 0.0001), less nervous (*M* difference = −0.69, *p* < 0.0001), less jittery (*M* difference = −0.90, *p* < 0.0001), and less irritable (*M* difference = −0.82, *p* < 0.0001), while simultaneously feeling more relaxed (*M* difference = +1.81, *p* < 0.0001), more inspired (*M* difference = +0.93, *p* < 0.0001), more rejuvenated (*M* difference = +0.39, *p* < 0.0001), and more calm (*M* difference = +0.35, *p* < 0.0001). Pre-post ratings of all affect terms are summarized in Table 1.

While Wilcoxon tests do not indicate magnitude, descriptive statistics provide some insight as to how large these increases and decreases were. For example, in a comparison of medians, the total PANAS-SF score for attendees increased by a median of 6 points (the median was 12 points before viewing the installation and 18 points after the experience). To contextualize numbers, the PANAS scale baseline for students being asked about their mental state in the current moment is 14.9 points. This means the median attendee in this sample entered the installation with a lower positive affect score than average and left with a higher positive affect score than average (Watson et al., 1988). However, further research would be required to fully quantify the emotional impact of immersive art exhibits and determine the significance of that magnitude. At present, these results affirm that the direction of the change is consistently in line with our hypothesis and the fact and direction of the affect change is statistically significant.

The post-visit survey included open-ended questions that provided visitors a chance to reflect on the experience. Visitors described the exhibit as “calming,” “relaxing,” and “soothing,” with participants often emphasizing its ability to promote “stress relief,” “peace,” and “tranquility.” Common descriptions highlighted the multisensory nature of the experience, incorporating “visual imagery,” “music,” and the “unique architecture of the space” to create an environment conducive to “mindfulness” and “reflection.” Qualitative responses confirmed the quantitative findings and added texture to our understanding of the impact of the experience on visitors.

Qualitative feedback addressed the following categories:

- *Immersive environment*: participants felt as though they were submerged in an environment, often likened to space, the ocean, or a planetarium, offering an “out of this world” sensation.
- *Stress relief and relaxation*: many noted that the experience provided a space to “turn off your brain,” reduce stress, and “return to factory settings.” The calming effects were often compared to meditation or mindfulness practices.
- *Visual and auditory appeal*: the projections of natural elements like waves, stars, and jellyfish, paired with soothing music, were repeatedly mentioned as awe-inspiring and mesmerizing.
- *Emotional impact*: several participants mentioned feeling inspired, rejuvenated, and connected, with one stating it “penetrates your soul” and rekindles joy in life’s beauty.
- *Unique setting*: the use of a chapel enhanced the experience, with participants noting the thoughtful integration of light, sound, and architecture.
- *Varied reactions*: while most reactions were positive, some found the technology simple, describing it as a “chill” light show rather than groundbreaking, but still appreciated the calming atmosphere and one respondent shared that initially it felt a bit overwhelming “At first I was a little overwhelmed but you start to settle in and take a couple of deep breaths and close your eyes… I started to feel much better.”

## 4 Discussion

This pilot study highlights the potential of immersive art installations to improve mood and reduce stress within a university setting, particularly during high-pressure periods such as midterms or final examinations. The statistically significant increases in positive affects (e.g., relaxation, inspiration, rejuvenation) and decreases in negative effects (e.g., distress, nervousness, irritability) after interacting with the immersive art installation suggest that exposure to immersive art may offer emotional benefits to learners and others who work in college settings. The immersive nature of the installation, which engaged multiple senses through carefully curated visual and auditory elements, likely contributed to this impact. Unlike static artworks that may be observed briefly and from a distance, the immersive art exhibit encouraged prolonged exposure to the work, fostering a deeper engagement and potentially facilitating mindfulness—a known mediator of stress reduction and emotional regulation. The setting of the pilot study—a university during final examination week—emphasizes the relevance of immersive art installations as a tool for promoting mental health in high-stress environments.

The open-ended responses provided by participants further support the benefit of installations on campuses. Comments such as “a space to allow your brain to return to factory settings” and “very calming and soothing” illustrate the perceived restorative effects of the experience. Additionally, respondents’ suggestions to make such installations available year-round reflect the ongoing demand for accessible opportunities for “relaxation” and “calming down” during times of heightened stress. The Healthy Minds survey data highlights the alarming prevalence of depressive symptoms, anxiety, and even suicidal ideation among college students (Lipson et al., 2023). This context underscores the urgency of exploring and implementing novel interventions to support learners and other members of the college community, such as this immersive art installation, to address these challenges.

### 4.1 Strengths and limitations

One of the pilot study’s strengths is its mixed-methods approach, which combined quantitative data from the PANAS survey with qualitative feedback to provide a more comprehensive understanding of the immersive art installation’s impact. The high response rate (over 90%) also lends credibility to the findings.

Despite the present findings, there are a number of limitations to this study. This was a pilot study and we deployed a convenience sample approach. Convenience samples may not be representative of the broader population, which limits the generalizability of the findings. While the results indicate changes in mood, the study does not account for the potential influence of external factors, such as prior exposure to art or variations in individual susceptibility to sensory stimuli. The current pilot study also does not account for variations in internal factors, such as coping style. The duration of the effects of the art installation remain unknown, as the data only captures immediate pre- and post-visit emotions. Causal attributions of these temporary shifts in affect cannot be made. Further, no attributions or assumptions can be made regarding the impact of viewing the art installation on specific stress triggers for example, final-exam related stress. Longitudinal studies would be beneficial to determine whether the positive emotional changes observed persist beyond the experience, along with the duration of these changes.

Furthermore, while the descriptive statistics suggest meaningful changes in affect, the pilot study did not quantify the magnitude of these changes beyond statistical significance. Additionally, while the questionnaire asked respondents to focus on exposure to the installation, it is not clear if, or to what extent changes in mood are associated with spending time experiencing the exhibit with friends, peers and colleagues. Future research could explore the use of additional scales or tools to measure the depth and duration of emotional shifts and limit the impact of other variables on the findings. Specifically, measures of stress and mood beyond a measurement of brief affect are needed. It is unknown the degree to which participants were directly impacted by the stress of end-of-term/exam season. Rather, PANAS scores reflected general *in vivo* levels of negative affectivity.

### 4.2 Conclusions and future directions

This pilot study provides evidence that immersive art installations have the potential to be effective interventions to promote emotional wellbeing, particularly in high-stress environments like college campuses. By engaging viewers in a multisensory experience, immersive art offers a novel way to foster relaxation, reduce stress, and improve mood. As universities continue to prioritize mental health, incorporating immersive art into their strategies may provide a creative and impactful solution to support students, faculty, and staff. Areas for future research may include the addition of psychophysiological measures of stress during viewing of immersive art installations, additional measures of mood and stress, and prospective and longitudinal data collection.

## Data Availability

The raw data supporting the conclusions of this article will be made available by the authors, without undue reservation.
