# Supplementary material for: “University stress” exploring the potential impact of an immersive art experience on a college campus
Source: Front Psychol. 2025 Nov 12;16:1592649. doi: 10.3389/fpsyg.2025.1592649 (PMC12646889; doi:10.3389/fpsyg.2025.1592649)
Supplement: Supplementary file 1 [file Data_Sheet_1.pdf]

Pre-visit Survey

Please complete the survey below before you enter the chapel.

Verbal Consent Statement Hello! Thank you for attending the "Immersive Art for Well-Being" exhibit.

We are asking attendees to complete a brief survey about their experiences today. You are being invited to participate in a research study titled "Immersive Art for Well-Being". This study is being done by Dr. Rosie Frasso from the Jefferson College of Population Health.

The purpose of this research study is to explore the value of art, like the interactive exhibit you'll be experiencing, as a tool to promote reflection and enhance education. If you agree to take part in this study, you will be asked to complete the survey/questionnaire on the next page. This survey/questionnaire will ask about the Immersive Art for Well-Being exhibit.

If you would rather not reflect on the exhibit, we completely understand and you do not need to proceed. However, if you are willing to do this, please note we expect this to take between 10 and 15 minutes to complete a pre- and post-visit survey.

You may not directly benefit from this research; however, we hope that your participation in the study may help inform future educational endeavors at Jefferson and in other settings. To the best of our ability, your answers in this study will remain confidential. We will minimize any risks to breach of confidentiality by not attaching your name to the survey.

Please note, your participation in this study is completely voluntary and you can withdraw at any time. You are free to skip any question you choose. As researchers, we are not qualified to provide counseling services and we will not be following up with you after this survey. If you feel upset after completing the survey, or find that some questions or aspects of the survey triggered distress, talking with a qualified clinician may help. If you feel you would like assistance, employees should contact Jefferson's Employee Work-Life Services at 215-955-8962 and students should reach out to the Student Personal Counseling Center at 215-955-HELP (4357) or (215) 503-2817 or (215) 951-2868.

If you have questions about this project or if you have a research-related problem, you may contact the researcher, Dr. Rosie Frasso at rosie.frasso@jefferson.edu. If you have any questions concerning your rights as a research subject, you may contact the Thomas Jefferson Institutional Review Board at 215-503-8966.

Which of the following best describes you? Select all that apply.

- ☐ Student
- ☐ Faculty member
- ☐ Staff member
- ☐ None of the Above

What brought you here today?

- ○ Invited
- ○ Saw a flyer on campus
- ○ I know the artist(s)
- ○ Course requirement
- ○ My community program invited me
- ○ Other

What do you hope to get out of this visit? Select all that apply.

- ☐ Enjoy art
- ☐ Relaxation
- ☐ Socialization
- ☐ Increase happiness
- ☐ Other

If you marked other, please elaborate:

____

To what extent do you feel wonder right now?

- ○ Very slightly or not at all
- ○ A little
- ○ Moderately
- ○ Quite a bit
- ○ Extremely

To what extent do you feel connected to others right now?

- ◯ Very slightly or not at all
- ◯ A little
- ◯ Moderately
- ◯ Quite a bit
- ◯ Extremely

To what extent do you feel relaxed right now?

- ◯ Very slightly or not at all
- ◯ A little
- ◯ Moderately
- ◯ Quite a bit
- ◯ Extremely

Indicate the extent to which you feel this way now:

	Very slightly or not at all	A little	Moderately	Quite a bit	Extremely
Interested	◯	◯	◯	◯	◯
Distressed	◯	◯	◯	◯	◯
Excited	◯	◯	◯	◯	◯
Upset	◯	◯	◯	◯	◯
Strong	◯	◯	◯	◯	◯
Guilty	◯	◯	◯	◯	◯
Scared	◯	◯	◯	◯	◯
Hostile	◯	◯	◯	◯	◯
Enthusiastic	◯	◯	◯	◯	◯
Proud	◯	◯	◯	◯	◯
Irritable	◯	◯	◯	◯	◯
Alert	◯	◯	◯	◯	◯
Ashamed	◯	◯	◯	◯	◯
Inspired	◯	◯	◯	◯	◯
Nervous	◯	◯	◯	◯	◯
Determined	◯	◯	◯	◯	◯
Attentive	◯	◯	◯	◯	◯
Jittery	◯	◯	◯	◯	◯
Active	◯	◯	◯	◯	◯
Afraid	◯	◯	◯	◯	◯

Describe your energy level right now. Select all that apply:

- ☐ Tired
- ☐ Energized
- ☐ Rejuvenated
- ☐ Calm

Post-visit Survey

Please complete the survey below after you leave the chapel.

Thank you!

Which word(s) best describe your experience in the exhibit? Select all that apply:	☐ Beautiful<br>☐ Scary<br>☐ Relaxing<br>☐ Sensory overload<br>☐ Joyous<br>☐ Stressful<br>☐ Awe-inspiring<br>☐ Overwhelming<br>☐ Calming
How long did you spend in the chapel?	◯ 0-15 minutes<br>◯ 15-30 minutes<br>◯ 30-45 minutes<br>◯ 45-60 minutes<br>◯ Over 60 minutes
Who might benefit from visiting this space?	____________________
Would you recommend a friend visit this installation? Why or why not?	____________________
Are there particular times during the semester (or year) that a visit to this space would be beneficial?	____________________
How would you describe this experience to a peer?	____________________
Would you encourage others to visit? What would you tell them?	____________________
Would you return to this space if it were available year-round?	◯ Yes<br>◯ No
To what extent do you feel wonder right now?	◯ Very slightly or not at all<br>◯ A little<br>◯ Moderately<br>◯ Quite a bit<br>◯ Extremely
To what extent do you feel connected to others right now?	◯ Very slightly or not at all<br>◯ A little<br>◯ Moderately<br>◯ Quite a bit<br>◯ Extremely

To what extent do you feel relaxed right now?

- ◯ Very slightly or not at all
- ◯ A little
- ◯ Moderately
- ◯ Quite a bit
- ◯ Extremely

Indicate the extent to which you feel this way now:

	Very slightly or not at all	A little	Moderately	Quite a bit	Extremely
Interested	◯	◯	◯	◯	◯
Distressed	◯	◯	◯	◯	◯
Excited	◯	◯	◯	◯	◯
Upset	◯	◯	◯	◯	◯
Strong	◯	◯	◯	◯	◯
Guilty	◯	◯	◯	◯	◯
Scared	◯	◯	◯	◯	◯
Hostile	◯	◯	◯	◯	◯
Enthusiastic	◯	◯	◯	◯	◯
Proud	◯	◯	◯	◯	◯
Irritable	◯	◯	◯	◯	◯
Alert	◯	◯	◯	◯	◯
Ashamed	◯	◯	◯	◯	◯
Inspired	◯	◯	◯	◯	◯
Nervous	◯	◯	◯	◯	◯
Determined	◯	◯	◯	◯	◯
Attentive	◯	◯	◯	◯	◯
Jittery	◯	◯	◯	◯	◯
Active	◯	◯	◯	◯	◯
Afraid	◯	◯	◯	◯	◯

Describe your energy level right now. Select all that apply:

- ☐ Tired
- ☐ Energized
- ☐ Rejuvenated
- ☐ Calm

Is there anything else you would like to share about your experience today?

____
